# Crystal structure of 4-methyl-*N*-propyl­benzene­sulfonamide

**DOI:** 10.1107/S2056989020007756

**Published:** 2020-06-12

**Authors:** Brock A. Stenfors, Rachel C. Collins, Jonah R. J. Duran, Richard J. Staples, Shannon M. Biros, Felix N. Ngassa

**Affiliations:** a Department of Chemistry, Grand Valley State University, 1 Campus Dr., Allendale, MI 49401, USA; b Center for Crystallographic Research, Department of Chemistry, Michigan State University, East Lansing, MI 48824, USA

**Keywords:** crystal structure, sulfonamide, inter­molecular N—H⋯O hydrogen bonding, inter­molecular C—H⋯O hydrogen bonding

## Abstract

The crystal structure of the title sulfonamide, C_10_H_15_NO_2_S, comprises two mol­ecules in the asymmetric unit. The S=O bond lengths of the sulfonamide functional group range from 1.428 (2) to 1.441 (2) Å, with S—C bond lengths of 1.766 (3) Å (for both mol­ecules in the asymmetric unit), and S—N bond lengths of 1.618 (2) and 1.622 (3) Å, respectively. When both mol­ecules are viewed down the N—S bond, the propyl group is *gauche* to the toluene moiety. In the crystal structure, mol­ecules of the title compound are arranged in an intricate three-dimensional network that is formed *via* inter­molecular C—H⋯O and N—H⋯O hydrogen bonds. The crystal structure was refined from a crystal twinned by inversion.

## Abstract 2

The title compound comprises two mol­ecules in the asymmetric unit. Inter­molecular C—H⋯O and N—H⋯O hydrogen bonds lead to a three-dimensional network structure.

## Chemical context   

Mol­ecules containing the sulfonamide moiety are found among a variety of biologically significant compounds, and have been used to inhibit a variety of enzymes to improve or repair biological functions. Commonly referred to as ‘sulfa drugs’, these mol­ecules have been in clinical use since 1968 (Connor, 1998 ▸). Since then, many sulfonamides have been recognized as effective inhibitors of the zinc enzyme carbonic anhydrase (Gul *et al.*, 2018 ▸). Several inter­esting anti­cancer properties are exhibited upon inhibition of this enzyme (Supuran *et al.*, 2001 ▸).
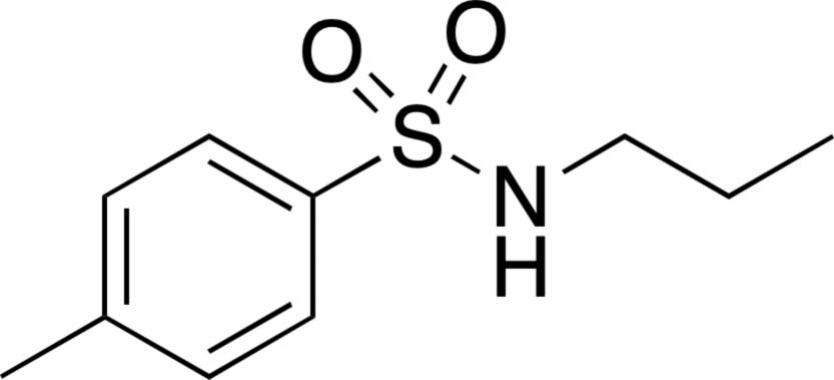


**Figure 1:**
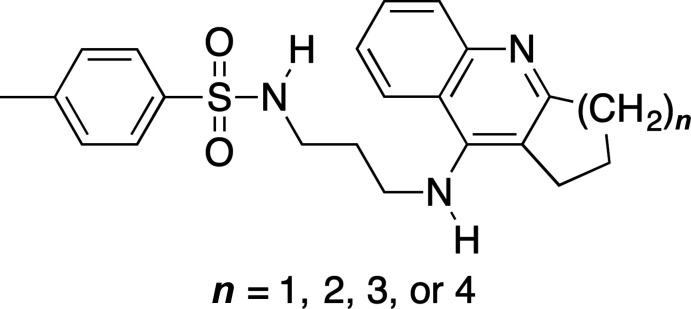
Acetyl­cholinesterase (AChE) and butyrylcholinesterase (BChE) inhibitors containing the *N*-propyl-4-methyl­benzene­sulfonamide moiety.

The title compound, 4-methyl-*N*-propyl­benzene­sulfon­amide, is structurally similar to a variety of biologically significant compounds. In particular, tacrine-*p*-toluene­sulfon­amide derivatives containing the 4-methyl-*N*-propyl­benzene­sulfonamide moiety have proven to be effective acetyl­cholinesterase (AChE) and butyrylcholinesterase (BChE) inhibitors (Makhaeva *et al.*, 2019 ▸; Fig. 1 ▸). The AChE cholinesterase enzyme catalyzes the hydrolysis of acetyl­cho­line (ACh), a neurotransmitter with the ability to coordinate neural responses in the brain (Picciotto *et al.*, 2012 ▸). The inhibition of AChE decreases the extent of ACh hydrolysis and enhances cholinergic transmission. AChE inhibition treats the symptoms of neuron deterioration characteristic of Alzheimer’s disease (García-Ayllón *et al.*, 2011 ▸). While BChE and AChE both regulate the cholinergic system, the effects of BChE are more prevalent in the blood than the nervous system (Pohanka, 2014 ▸). BChE is, however, found in the central nervous system and is involved in the formation or growth of β-amyloid plaques (Kim *et al.*, 2016 ▸). The inhibition of both AChE and BChE improves cognitive function and minimizes the accumulation of β-amyloid and is a viable strategy for treating Alzheimer’s disease.

A facile synthesis of sulfonamides is necessary to produce a variety of compounds with the potential to improve human health. A review of the current literature suggests that nucleophilic substitution of sulfonyl halides or sulfonic acids with an amine is an efficient method for the synthesis of sulfonamides (Mukherjee *et al.*, 2018 ▸; De Luca & Giacomelli, 2008 ▸). The title compound was synthesized by reacting *p*-toluene­sulfonyl chloride with propyl­amine in the presence of pyridine. The reaction was carried out in an inert atmosphere, using di­chloro­methane as the solvent. These reaction conditions resulted in a poor yield and slow reaction time. To work toward a facile synthesis of sulfonamides, a more efficient and environmentally benign method was recently developed. By substituting pyridine and di­chloro­methane with aqueous potassium carbonate and tetra­hydro­furan, a significant increase in the yield and rate of the reaction was observed. The products formed under these reaction conditions are easily isolated upon acidification of the reaction mixture. Furthermore, the solvent combination supports a broader range of nitro­gen nucleophiles. In our ongoing efforts to synthesize and characterize sulfonamide products, the synthesis and crystal structure of 4-methyl-*N*-propyl­benzene­sulfonamide is reported here.

## Structural commentary   

**Figure 2:**
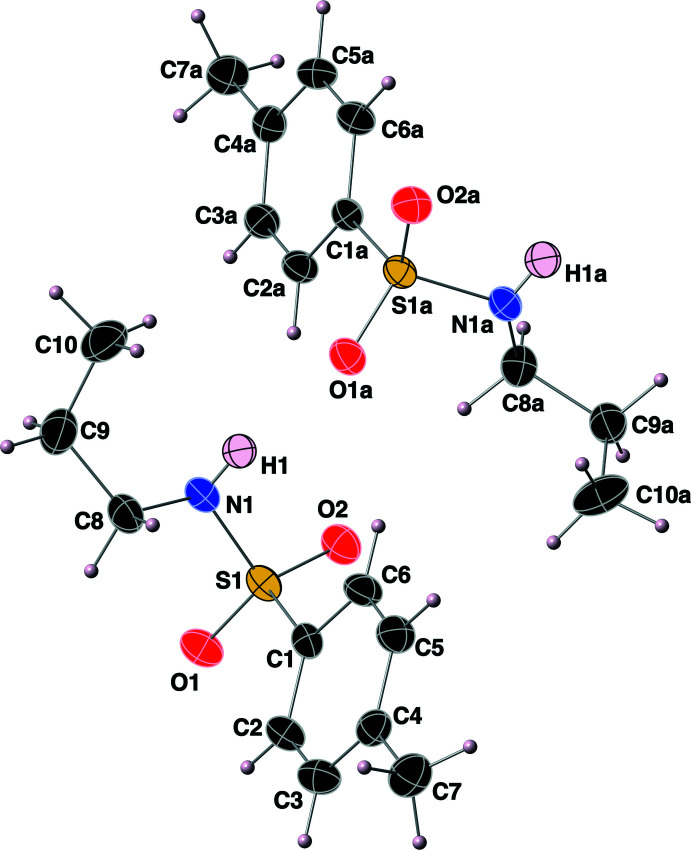
The structures of the two mol­ecules in the asymmetric unit of the title compound, with the atom-labeling scheme. Displacement ellipsoids are shown at the 40% probability level using standard CPK colors.

**Figure 3:**
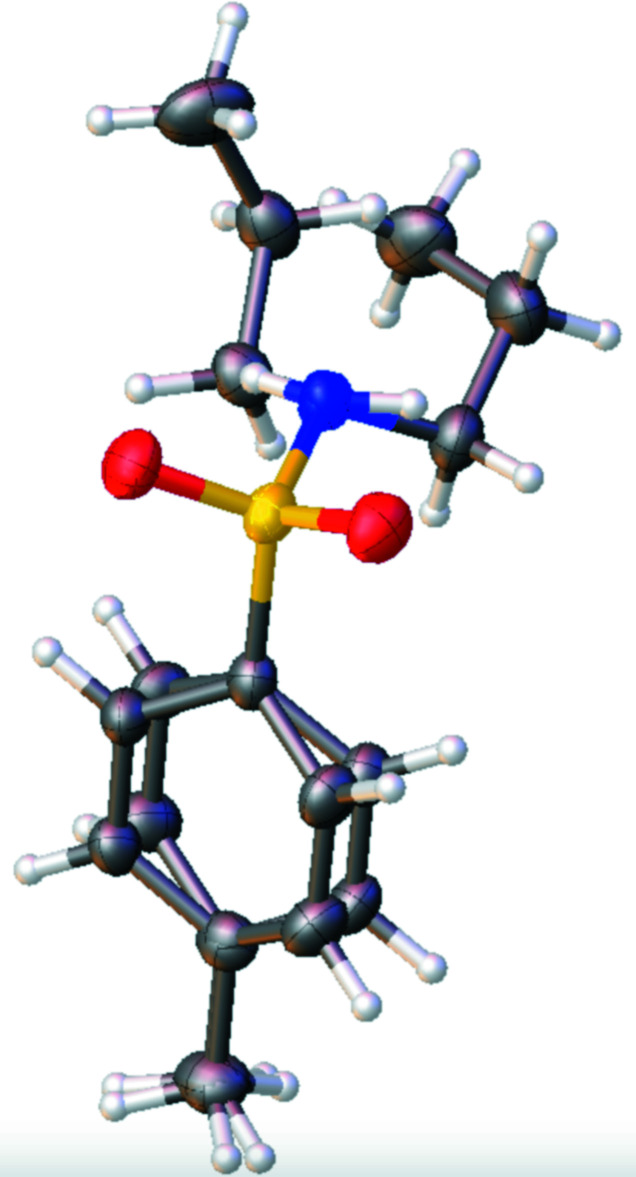
Overlay plot of the two independent mol­ecules in the title compound, with grouping of the atoms C1—S1—-N1 and C1*A*—S1*A*—N1*A*, and the mol­ecule oriented so as to view it down the S—N bond. Displacement ellipsoids are as in Fig. 2 ▸.

The title compound comprises two equivalents of the mol­ecule in the asymmetric unit, as shown in Fig. 2 ▸ (suffix ‘*A*′ for all atomic labels used for the second mol­ecule). The S=O bond lengths of the sulfonamide functional group range from 1.428 (2) to 1.441 (2) Å, which fall within expected values. The S—C bond lengths are 1.766 (3) Å for both mol­ecules, and the S—N bond lengths are 1.618 (2) and 1.622 (3) Å. The O—S—O bond angles are 119.49 (13) and 118.26 (13)°, with N—S—C bond angles of 106.86 (13) and 108.27 (13)°. The two independent mol­ecules differ in the orientation of the propyl chain and the H atom attached to the N atom, however, in each case with the propyl chain being *gauche* to a sulfonamide oxygen atom and to the toluene moiety when the mol­ecules are viewed down the N1—S1 bond (Fig. 3 ▸). The torsion angles between the first carbon atom of the propyl chain (C8 or C8*A*) and the sulfonamide oxygen atom O1 or O1*A* are 60.5 (3) and 57.3 (2)°, respectively. The groups bonded to the sulfur atom of both sulfonamide groups adopt slightly distorted tetra­hedral environments with fourfold coordination τ_4_ descriptors of 0.94 for both S1 and S1*A* (ideal values are 0 for square-planar, 0.85 for trigonal pyramidal, and 1 for tetra­hedral coordinations; Yang *et al.*, 2007 ▸).

## Supra­molecular features   

**Figure 4:**
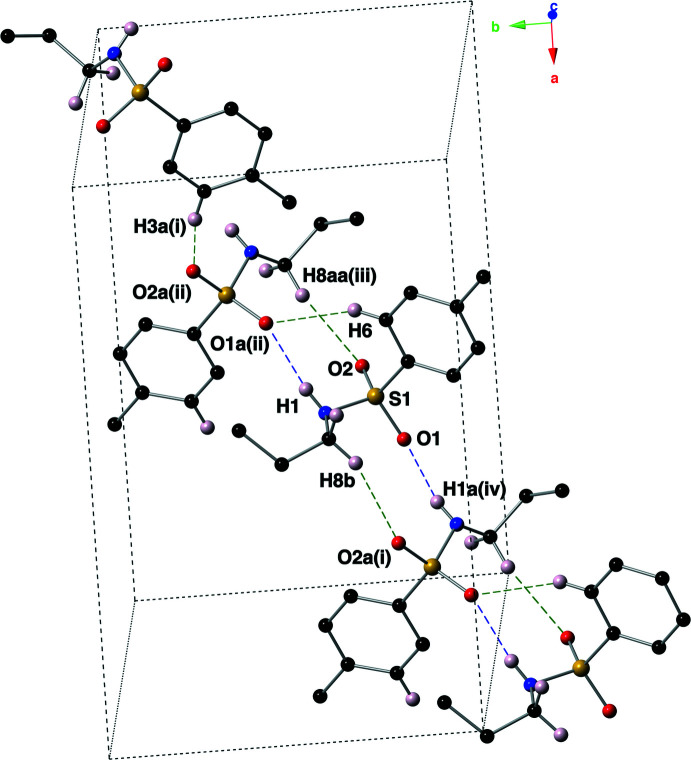
A diagram showing the specific hydrogen-bonding inter­actions (N—H⋯O: purple dashed lines, C—H⋯O: green dashed lines) present in the title compound, using a ball-and-stick model with standard CPK colors. Hydrogen atoms bonded to parent atoms that are not involved in a non-covalent inter­action have been omitted for clarity. [Symmetry codes: (i) *x* + 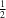, −*y* + 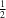, *z* + 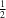; (ii) *x*, −*y* + 1, *z* + 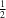; (iii) *x*, −*y* + 1, *z* − 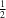; (iv) *x* − 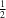, −*y* + 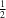, *z* − 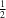].

**Figure 5:**
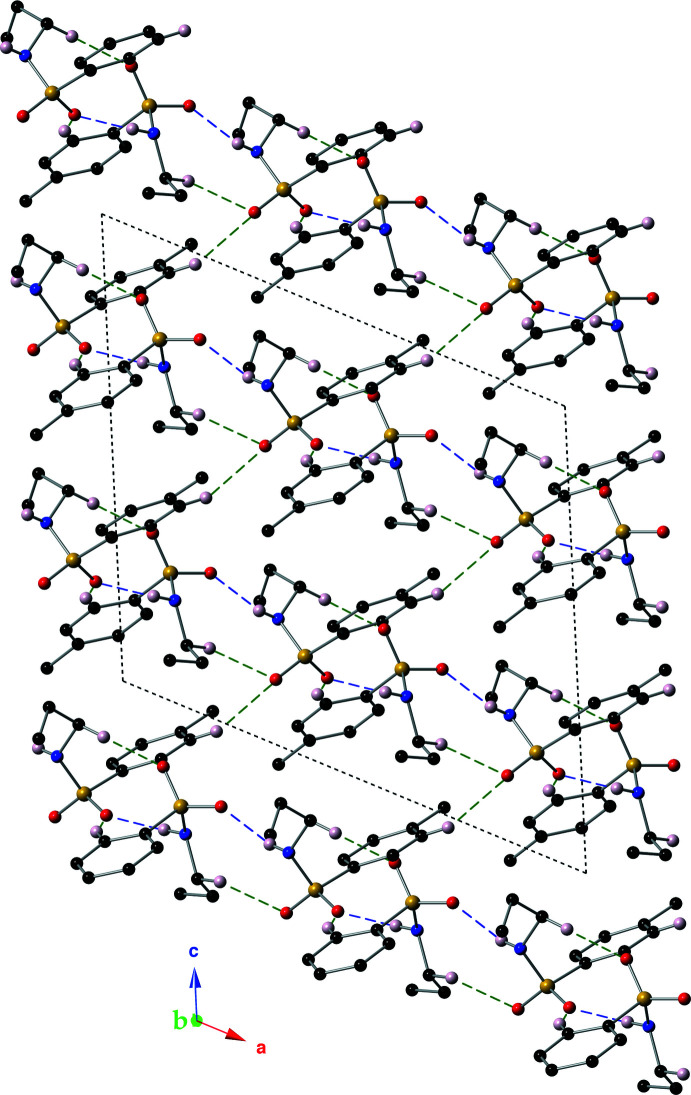
A packing diagram of the title compound viewed down the *b* axis. Inter­molecular hydrogen bonds are shown with dashed lines (N—H⋯O: purple, C—H⋯O: green). This figure was drawn using a ball and stick model with standard CPK colors. Hydrogen atoms bonded to parent atoms that are not involved in a non-covalent inter­action have been omitted for clarity.

Hydrogen-bonding inter­actions, both N—H⋯O and C—H⋯O, hold mol­ecules of the title compound together in the crystal structure (Table 1 ▸, Fig. 4 ▸). The inter­molecular N—H⋯O inter­actions are between the sulfonamide N(H) atoms and the oxygen (O1 or O1*A*) atoms of a nearby mol­ecule. These classic hydrogen-bonding inter­actions form ribbons of the title compound that lie parallel to the *ab* plane. These inter­actions have *D*⋯*A* distances of 2.925 (3) and 2.968 (3) Å, with *D*—H⋯*A* angles of 161 (3) and 172 (3)°. The inter­molecular C—H⋯O hydrogen bonding inter­actions (Sutor, 1958 ▸, 1962 ▸, 1963 ▸; Steiner, 1996 ▸) have, as expected, longer *D*⋯*A* distances ranging from 3.399 (4) to 3.594 (4) Å, and *D*—H⋯*A* angles ranging from 152.8 to 170.2°. Specific­ally, the C8(H8*B*)⋯O2*A*, C8*A*(H8*AA*)⋯O2 and C6(H6)⋯O1*A* inter­actions contribute to the stabilization of the supra­molecular ribbons. The inter­action between C3*A*(H3*A*) and O2*A* links the supra­molecular ribbons into an intricate three-dimensional network (Fig. 5 ▸).

## Database survey   

A search for structures containing the *p*-methyl­benzene­sulfonamide entity in the Cambridge Structural Database (CSD, Version 5.41, November, 2019; Groom *et al.*, 2016 ▸), where the nitro­gen atom bears one carbon-containing group, resulted in over 2,200 hits. A few structures with relatively simple, yet inter­esting, –*R* groups bonded to the sulfonamide nitro­gen atom are BOLPOH (Germain *et al.*, 1983 ▸), AZUQUI (Rehman *et al.*, 2011 ▸), AYURUI and AYURUI01 (Khan *et al.*, 2011 ▸; Akyıldız *et al.*, 2018 ▸), and ATOVIO (Muller *et al.*, 2004 ▸). In the structures of BOLPOH and AZUQUI, the –*R* groups are both aromatic systems with a quinoline ring and a 4-amino­benzene ring, respectively. The structures of AYURUI and AYURUI01 contain two *p*-methyl­benzene­sulfonamide groups linked *via* a propane chain. Lastly, the –*R* group in ATOVIO is a tri­cyclo­heptyl ring system.

## Synthesis and crystallization   

The title compound was prepared by the dropwise addition of 0.59 *M* aqueous potassium carbonate (10 ml, 5.90 mmol) to a stirring mixture of propyl­amine (0.49 ml, 5.90 mmol) and *p*-toluene­sulfonyl chloride (1.00 g, 5.25 mmol) in 10 ml of tetra­hydro­furan. The reaction mixture was stirred at room temperate for 24 h under a nitro­gen atmosphere. After acidification with 5 *M* HCl and dilution with 15 ml of di­chloro­methane, the organic layer was washed with water and brine. The aqueous layers were back extracted with 10 ml of di­chloro­methane. The combined organic layers were then combined, dried over anhydrous sodium sulfate, and evaporated to dryness. The liquid residue was triturated with diethyl ether, placed in a freezer for 48 h and, after isolation *via* vacuum filtration, the product was obtained as colorless crystals (59%; m.p. 335–337 K).

## Refinement   

Crystal data, data collection and structure refinement details are summarized in Table 2 ▸. The crystal under investigation was twinned by inversion, with a refined Flack parameter of 0.443 (19) (Parsons *et al.*, 2013 ▸). For this structure, hydrogen atoms bonded to carbon atoms were placed in calculated positions and refined to ride on their parent atoms: C—H = 0.95–1.00 Å with *U*_iso_(H) = 1.2*U*_eq_(C) for methyl­ene groups and aromatic hydrogen atoms, and *U*_iso_(H) = 1.5*U*_eq_(C) for methyl groups. Hydrogen atoms bonded to nitro­gen atoms were located using electron density difference maps, and were refined freely.

## Supplementary Material

Crystal structure: contains datablock(s) I. DOI: 10.1107/S2056989020007756/wm5567sup1.cif

Structure factors: contains datablock(s) I. DOI: 10.1107/S2056989020007756/wm5567Isup2.hkl

Supporting information file. DOI: 10.1107/S2056989020007756/wm5567Isup3.cml

CCDC reference: 2008411

Additional supporting information:  crystallographic information; 3D view; checkCIF report

## Figures and Tables

**Table 1:** Hydrogen-bond geometry (Å, °)

*D*—H⋯*A*	*D*—H	H⋯*A*	*D*⋯*A*	*D*—H⋯*A*
C3*A*—H3*A*⋯O2*A* ^i^	0.95	2.53	3.399 (4)	153
C6—H6⋯O1*A* ^ii^	0.95	2.59	3.474 (4)	156
C8—H8*B*⋯O2*A* ^i^	0.99	2.56	3.489 (4)	156
C8*A*—H8*AA*⋯O2^iii^	0.99	2.61	3.594 (4)	170
N1*A*—H1*A*⋯O1^iv^	0.82 (3)	2.14 (3)	2.925 (3)	161 (3)
N1—H1⋯O1*A* ^ii^	0.85 (3)	2.13 (4)	2.968 (3)	172 (3)

Symmetry codes: (i) 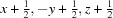; (ii) 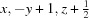; (iii) 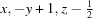; (iv) 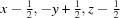.

**Table 2:** Experimental details

Crystal data	
Chemical formula	C_10_H_15_NO_2_S
*M*_r_	213.29
Crystal system, space group	Monoclinic, *C* *c*
Temperature (K)	173
*a*, *b*, *c* (Å)	15.9353 (9), 10.3526 (6), 14.8486 (9)
β (°)	115.1347 (6)
*V* (Å^3^)	2217.7 (2)
*Z*	8
Radiation type	Mo *K*α
μ (mm^−1^)	0.27
Crystal size (mm)	0.45 × 0.40 × 0.39

Data collection	
Diffractometer	Bruker APEXII CCD
Absorption correction	Multi-scan (*SADABS*; Bruker, 2013 ▸)
*T*_min_, *T*_max_	0.684, 0.745
No. of measured, independent and observed [*I* > 2σ(*I*)] reflections	18931, 4554, 4422
*R*_int_	0.028
(sin θ/λ)_max_ (Å^−1^)	0.626

Refinement	
*R*[*F* ^2^ > 2σ(*F* ^2^)], *wR*(*F* ^2^), *S*	0.031, 0.083, 1.02
No. of reflections	4554
No. of parameters	265
No. of restraints	2
H-atom treatment	H atoms treated by a mixture of independent and constrained refinement
Δρ_max_, Δρ_min_ (e Å^−3^)	0.27, −0.20
Absolute structure	Flack *x* determined using 2140 quotients [(*I* ^+^)−(*I* ^−^)]/[(*I* ^+^)+(*I* ^−^)] (Parsons *et al.*, 2013 ▸)
Absolute structure parameter	0.443 (19)

Computer programs: *APEX2* and *SAINT* (Bruker, 2013 ▸), *SHELXS* (Sheldrick, 2008 ▸), *SHELXL* (Sheldrick, 2015 ▸), *OLEX2* (Dolomanov *et al.*, 2009 ▸; Bourhis *et al.*, 2015 ▸) and *CrystalMaker* (Palmer, 2007 ▸).

## supplementary crystallographic information

### Crystal data

**Table:**

C_10_H_15_NO_2_S	*F*(000) = 912
*M**_r_* = 213.29	*D*_x_ = 1.278 Mg m^−^^3^
Monoclinic, *C**c*	Mo *K*α radiation, λ = 0.71073 Å
*a* = 15.9353 (9) Å	Cell parameters from 9996 reflections
*b* = 10.3526 (6) Å	θ = 2.4–26.4°
*c* = 14.8486 (9) Å	µ = 0.27 mm^−^^1^
β = 115.1347 (6)°	*T* = 173 K
*V* = 2217.7 (2) Å^3^	Block, colourless
*Z* = 8	0.45 × 0.40 × 0.39 mm

### Data collection

**Table:**

Bruker APEXII CCD diffractometer	4422 reflections with *I* > 2σ(*I*)
φ and ω scans	*R*_int_ = 0.028
Absorption correction: multi-scan (*SADABS*; Bruker, 2013)	θ_max_ = 26.4°, θ_min_ = 2.4°
*T*_min_ = 0.684, *T*_max_ = 0.745	*h* = −19→19
18931 measured reflections	*k* = −12→12
4554 independent reflections	*l* = −18→18

### Refinement

**Table:**

Refinement on *F*^2^	Hydrogen site location: mixed
Least-squares matrix: full	H atoms treated by a mixture of independent and constrained refinement
*R*[*F*^2^ > 2σ(*F*^2^)] = 0.031	*w* = 1/[σ^2^(*F*_o_^2^) + (0.0503*P*)^2^ + 0.9882*P*] where *P* = (*F*_o_^2^ + 2*F*_c_^2^)/3
*wR*(*F*^2^) = 0.083	(Δ/σ)_max_ < 0.001
*S* = 1.02	Δρ_max_ = 0.27 e Å^−^^3^
4554 reflections	Δρ_min_ = −0.20 e Å^−^^3^
265 parameters	Absolute structure: Flack *x* determined using 2140 quotients [(*I*^+^)-(*I*^-^)]/[(*I*^+^)+(*I*^-^)] (Parsons *et al.*, 2013)
2 restraints	Absolute structure parameter: 0.443 (19)
Primary atom site location: structure-invariant direct methods	

### Special details

**Table:**

Geometry. All esds (except the esd in the dihedral angle between two l.s. planes) are estimated using the full covariance matrix. The cell esds are taken into account individually in the estimation of esds in distances, angles and torsion angles; correlations between esds in cell parameters are only used when they are defined by crystal symmetry. An approximate (isotropic) treatment of cell esds is used for estimating esds involving l.s. planes.

### Fractional atomic coordinates and isotropic or equivalent isotropic displacement parameters (Å^2^)

**Table:**

	*x*	*y*	*z*	*U*_iso_*/*U*_eq_	
S1A	0.40337 (4)	0.32398 (6)	0.21849 (4)	0.02809 (16)	
S1	0.61545 (4)	0.30322 (7)	0.77858 (4)	0.03083 (17)	
O2A	0.33689 (14)	0.2460 (2)	0.14206 (15)	0.0368 (5)	
C1A	0.48935 (19)	0.2218 (3)	0.3034 (2)	0.0266 (5)	
C5	0.3803 (2)	0.1709 (3)	0.5601 (3)	0.0388 (7)	
H5	0.3189	0.2015	0.5231	0.047*	
C3A	0.6417 (2)	0.1911 (3)	0.4327 (2)	0.0325 (6)	
H3A	0.7009	0.2249	0.4749	0.039*	
C1	0.5341 (2)	0.2024 (3)	0.6871 (2)	0.0300 (6)	
C6	0.4440 (2)	0.2486 (3)	0.6334 (2)	0.0366 (7)	
H6	0.4267	0.3316	0.6470	0.044*	
C4	0.4047 (2)	0.0488 (3)	0.5397 (2)	0.0347 (6)	
C2A	0.5763 (2)	0.2713 (3)	0.3643 (2)	0.0324 (6)	
H2A	0.5905	0.3593	0.3590	0.039*	
N1	0.62264 (18)	0.4342 (2)	0.72284 (18)	0.0328 (5)	
O2	0.58027 (16)	0.3406 (2)	0.84936 (15)	0.0400 (5)	
C4A	0.6225 (2)	0.0614 (3)	0.4409 (2)	0.0319 (6)	
O1A	0.45006 (14)	0.4257 (2)	0.19202 (16)	0.0362 (5)	
C2	0.5590 (2)	0.0820 (3)	0.6681 (2)	0.0370 (7)	
H2	0.6204	0.0514	0.7050	0.044*	
C3	0.4942 (2)	0.0052 (3)	0.5949 (2)	0.0418 (7)	
H3	0.5115	−0.0784	0.5825	0.050*	
N1A	0.34797 (17)	0.3914 (2)	0.27586 (19)	0.0332 (5)	
C5A	0.5348 (2)	0.0145 (3)	0.3788 (2)	0.0350 (6)	
H5A	0.5204	−0.0736	0.3837	0.042*	
C8	0.6443 (2)	0.4213 (3)	0.6357 (2)	0.0399 (7)	
H8A	0.5885	0.3916	0.5774	0.048*	
H8B	0.6936	0.3558	0.6499	0.048*	
C9A	0.3349 (2)	0.5714 (3)	0.3760 (2)	0.0421 (7)	
H9AA	0.2776	0.5292	0.3720	0.051*	
H9AB	0.3659	0.6119	0.4425	0.051*	
C6A	0.4685 (2)	0.0937 (3)	0.3105 (2)	0.0338 (6)	
H6A	0.4090	0.0604	0.2688	0.041*	
O1	0.70368 (15)	0.2382 (2)	0.81488 (17)	0.0411 (5)	
C7	0.3343 (3)	−0.0346 (3)	0.4595 (3)	0.0480 (8)	
H7A	0.3372	−0.0177	0.3960	0.072*	
H7B	0.2720	−0.0144	0.4535	0.072*	
H7C	0.3480	−0.1259	0.4772	0.072*	
C7A	0.6943 (2)	−0.0253 (3)	0.5154 (2)	0.0420 (7)	
H7AA	0.7558	0.0133	0.5359	0.063*	
H7AB	0.6928	−0.1099	0.4851	0.063*	
H7AC	0.6810	−0.0358	0.5737	0.063*	
C8A	0.3984 (2)	0.4693 (3)	0.3654 (2)	0.0377 (6)	
H8AA	0.4523	0.5114	0.3608	0.045*	
H8AB	0.4219	0.4127	0.4248	0.045*	
C10	0.6052 (3)	0.6546 (4)	0.5879 (3)	0.0575 (10)	
H10D	0.5466	0.6260	0.5343	0.086*	
H10E	0.6273	0.7319	0.5666	0.086*	
H10F	0.5953	0.6747	0.6472	0.086*	
C9	0.6761 (3)	0.5489 (4)	0.6120 (3)	0.0535 (9)	
H9A	0.7334	0.5760	0.6696	0.064*	
H9B	0.6916	0.5370	0.5546	0.064*	
C10A	0.3090 (3)	0.6751 (4)	0.2977 (3)	0.0613 (11)	
H10A	0.2820	0.6352	0.2316	0.092*	
H10B	0.3645	0.7240	0.3061	0.092*	
H10C	0.2636	0.7335	0.3044	0.092*	
H1A	0.307 (2)	0.343 (3)	0.277 (2)	0.034 (9)*	
H1	0.576 (2)	0.481 (3)	0.713 (2)	0.030 (8)*	

### Atomic displacement parameters (Å^2^)

**Table:**

	*U*^11^	*U*^22^	*U*^33^	*U*^12^	*U*^13^	*U*^23^
S1A	0.0225 (3)	0.0303 (3)	0.0288 (3)	−0.0027 (3)	0.0082 (3)	0.0011 (3)
S1	0.0278 (3)	0.0362 (3)	0.0270 (3)	0.0067 (3)	0.0102 (3)	−0.0013 (3)
O2A	0.0286 (10)	0.0384 (11)	0.0336 (10)	−0.0029 (9)	0.0039 (9)	−0.0020 (9)
C1A	0.0250 (13)	0.0287 (13)	0.0257 (13)	−0.0013 (10)	0.0104 (11)	0.0008 (11)
C5	0.0275 (15)	0.0389 (17)	0.0436 (18)	0.0017 (12)	0.0088 (14)	−0.0004 (13)
C3A	0.0274 (14)	0.0338 (16)	0.0327 (15)	−0.0037 (11)	0.0094 (13)	−0.0031 (11)
C1	0.0281 (14)	0.0353 (14)	0.0289 (14)	0.0030 (11)	0.0143 (12)	0.0011 (11)
C6	0.0304 (14)	0.0304 (15)	0.0451 (17)	0.0054 (12)	0.0124 (14)	−0.0022 (12)
C4	0.0403 (16)	0.0338 (15)	0.0327 (14)	−0.0044 (12)	0.0180 (13)	−0.0004 (12)
C2A	0.0275 (14)	0.0305 (14)	0.0362 (15)	−0.0043 (11)	0.0106 (12)	0.0009 (11)
N1	0.0309 (12)	0.0334 (12)	0.0349 (12)	0.0048 (10)	0.0147 (10)	−0.0028 (10)
O2	0.0442 (12)	0.0468 (12)	0.0318 (10)	0.0060 (10)	0.0188 (9)	−0.0014 (9)
C4A	0.0348 (15)	0.0362 (15)	0.0263 (13)	0.0020 (12)	0.0143 (12)	0.0023 (11)
O1A	0.0305 (10)	0.0367 (11)	0.0405 (11)	−0.0013 (8)	0.0141 (9)	0.0073 (9)
C2	0.0347 (15)	0.0360 (15)	0.0392 (16)	0.0100 (12)	0.0147 (13)	0.0005 (13)
C3	0.0485 (19)	0.0360 (16)	0.0412 (17)	0.0063 (14)	0.0193 (15)	−0.0034 (13)
N1A	0.0244 (11)	0.0343 (13)	0.0404 (13)	−0.0051 (10)	0.0133 (10)	−0.0042 (10)
C5A	0.0417 (17)	0.0272 (14)	0.0353 (14)	−0.0046 (12)	0.0156 (13)	0.0016 (11)
C8	0.0415 (16)	0.0430 (16)	0.0420 (16)	0.0048 (14)	0.0243 (14)	−0.0015 (14)
C9A	0.0377 (16)	0.0497 (19)	0.0421 (17)	−0.0032 (14)	0.0199 (14)	−0.0107 (14)
C6A	0.0323 (14)	0.0337 (14)	0.0323 (14)	−0.0075 (12)	0.0105 (12)	−0.0024 (11)
O1	0.0334 (11)	0.0448 (13)	0.0379 (11)	0.0111 (9)	0.0083 (9)	0.0001 (9)
C7	0.051 (2)	0.0457 (18)	0.0440 (18)	−0.0119 (15)	0.0171 (16)	−0.0088 (15)
C7A	0.0437 (18)	0.0407 (17)	0.0381 (16)	0.0047 (13)	0.0139 (14)	0.0081 (13)
C8A	0.0321 (15)	0.0457 (16)	0.0323 (14)	−0.0014 (13)	0.0109 (12)	−0.0053 (13)
C10	0.079 (3)	0.0418 (19)	0.060 (2)	−0.0037 (19)	0.038 (2)	0.0091 (16)
C9	0.053 (2)	0.058 (2)	0.062 (2)	−0.0094 (17)	0.0365 (19)	−0.0031 (18)
C10A	0.082 (3)	0.042 (2)	0.051 (2)	0.0113 (19)	0.021 (2)	−0.0126 (16)

### Geometric parameters (Å, º)

**Table:**

S1A—O2A	1.428 (2)	N1A—C8A	1.469 (4)
S1A—C1A	1.766 (3)	N1A—H1A	0.82 (3)
S1A—O1A	1.437 (2)	C5A—H5A	0.9500
S1A—N1A	1.622 (3)	C5A—C6A	1.381 (4)
S1—C1	1.766 (3)	C8—H8A	0.9900
S1—N1	1.618 (3)	C8—H8B	0.9900
S1—O2	1.438 (2)	C8—C9	1.509 (5)
S1—O1	1.441 (2)	C9A—H9AA	0.9900
C1A—C2A	1.392 (4)	C9A—H9AB	0.9900
C1A—C6A	1.382 (4)	C9A—C8A	1.518 (4)
C5—H5	0.9500	C9A—C10A	1.505 (6)
C5—C6	1.387 (5)	C6A—H6A	0.9500
C5—C4	1.393 (4)	C7—H7A	0.9800
C3A—H3A	0.9500	C7—H7B	0.9800
C3A—C2A	1.381 (4)	C7—H7C	0.9800
C3A—C4A	1.394 (4)	C7A—H7AA	0.9800
C1—C6	1.397 (4)	C7A—H7AB	0.9800
C1—C2	1.374 (4)	C7A—H7AC	0.9800
C6—H6	0.9500	C8A—H8AA	0.9900
C4—C3	1.385 (5)	C8A—H8AB	0.9900
C4—C7	1.512 (4)	C10—H10D	0.9800
C2A—H2A	0.9500	C10—H10E	0.9800
N1—C8	1.481 (4)	C10—H10F	0.9800
N1—H1	0.85 (3)	C10—C9	1.503 (6)
C4A—C5A	1.394 (4)	C9—H9A	0.9900
C4A—C7A	1.505 (4)	C9—H9B	0.9900
C2—H2	0.9500	C10A—H10A	0.9800
C2—C3	1.389 (5)	C10A—H10B	0.9800
C3—H3	0.9500	C10A—H10C	0.9800
			
O2A—S1A—C1A	108.49 (13)	N1—C8—H8A	109.5
O2A—S1A—O1A	119.48 (13)	N1—C8—H8B	109.5
O2A—S1A—N1A	105.96 (13)	N1—C8—C9	110.6 (3)
O1A—S1A—C1A	107.43 (13)	H8A—C8—H8B	108.1
O1A—S1A—N1A	106.77 (13)	C9—C8—H8A	109.5
N1A—S1A—C1A	108.27 (13)	C9—C8—H8B	109.5
N1—S1—C1	106.86 (13)	H9AA—C9A—H9AB	107.8
O2—S1—C1	109.52 (13)	C8A—C9A—H9AA	109.0
O2—S1—N1	106.43 (13)	C8A—C9A—H9AB	109.0
O2—S1—O1	118.26 (13)	C10A—C9A—H9AA	109.0
O1—S1—C1	107.03 (13)	C10A—C9A—H9AB	109.0
O1—S1—N1	108.23 (14)	C10A—C9A—C8A	113.1 (3)
C2A—C1A—S1A	120.0 (2)	C1A—C6A—H6A	120.3
C6A—C1A—S1A	119.4 (2)	C5A—C6A—C1A	119.5 (3)
C6A—C1A—C2A	120.6 (3)	C5A—C6A—H6A	120.3
C6—C5—H5	119.4	C4—C7—H7A	109.5
C6—C5—C4	121.2 (3)	C4—C7—H7B	109.5
C4—C5—H5	119.4	C4—C7—H7C	109.5
C2A—C3A—H3A	119.4	H7A—C7—H7B	109.5
C2A—C3A—C4A	121.2 (3)	H7A—C7—H7C	109.5
C4A—C3A—H3A	119.4	H7B—C7—H7C	109.5
C6—C1—S1	118.5 (2)	C4A—C7A—H7AA	109.5
C2—C1—S1	120.8 (2)	C4A—C7A—H7AB	109.5
C2—C1—C6	120.7 (3)	C4A—C7A—H7AC	109.5
C5—C6—C1	118.8 (3)	H7AA—C7A—H7AB	109.5
C5—C6—H6	120.6	H7AA—C7A—H7AC	109.5
C1—C6—H6	120.6	H7AB—C7A—H7AC	109.5
C5—C4—C7	120.5 (3)	N1A—C8A—C9A	110.1 (2)
C3—C4—C5	118.7 (3)	N1A—C8A—H8AA	109.6
C3—C4—C7	120.8 (3)	N1A—C8A—H8AB	109.6
C1A—C2A—H2A	120.4	C9A—C8A—H8AA	109.6
C3A—C2A—C1A	119.2 (3)	C9A—C8A—H8AB	109.6
C3A—C2A—H2A	120.4	H8AA—C8A—H8AB	108.2
S1—N1—H1	108 (2)	H10D—C10—H10E	109.5
C8—N1—S1	117.7 (2)	H10D—C10—H10F	109.5
C8—N1—H1	115 (2)	H10E—C10—H10F	109.5
C3A—C4A—C5A	118.3 (3)	C9—C10—H10D	109.5
C3A—C4A—C7A	120.8 (3)	C9—C10—H10E	109.5
C5A—C4A—C7A	120.9 (3)	C9—C10—H10F	109.5
C1—C2—H2	120.1	C8—C9—H9A	108.9
C1—C2—C3	119.7 (3)	C8—C9—H9B	108.9
C3—C2—H2	120.1	C10—C9—C8	113.5 (3)
C4—C3—C2	120.9 (3)	C10—C9—H9A	108.9
C4—C3—H3	119.5	C10—C9—H9B	108.9
C2—C3—H3	119.5	H9A—C9—H9B	107.7
S1A—N1A—H1A	111 (2)	C9A—C10A—H10A	109.5
C8A—N1A—S1A	120.07 (19)	C9A—C10A—H10B	109.5
C8A—N1A—H1A	117 (2)	C9A—C10A—H10C	109.5
C4A—C5A—H5A	119.4	H10A—C10A—H10B	109.5
C6A—C5A—C4A	121.2 (3)	H10A—C10A—H10C	109.5
C6A—C5A—H5A	119.4	H10B—C10A—H10C	109.5

### Hydrogen-bond geometry (Å, º)

**Table:**

*D*—H···*A*	*D*—H	H···*A*	*D*···*A*	*D*—H···*A*
C3*A*—H3*A*···O2*A*^i^	0.95	2.53	3.399 (4)	153
C6—H6···O1*A*^ii^	0.95	2.59	3.474 (4)	156
C8—H8*B*···O2*A*^i^	0.99	2.56	3.489 (4)	156
C8*A*—H8*AA*···O2^iii^	0.99	2.61	3.594 (4)	170
N1*A*—H1*A*···O1^iv^	0.82 (3)	2.14 (3)	2.925 (3)	161 (3)
N1—H1···O1*A*^ii^	0.85 (3)	2.13 (4)	2.968 (3)	172 (3)

Symmetry codes: (i) *x*+1/2, −*y*+1/2, *z*+1/2; (ii) *x*, −*y*+1, *z*+1/2; (iii) *x*, −*y*+1, *z*−1/2; (iv) *x*−1/2, −*y*+1/2, *z*−1/2.
